# Clinical Illustrations of the Diseases of India, as Exhibited in the Medical History of a Body of European Soldiers for a Series of Years from Their Arrival in That Country

**Published:** 1847-01

**Authors:** 


### Art VI.

*Clinical Illustrations of the Diseases of India, as exhibited in the Medical History of a Body of European Soldiers, for a series of years from their arrival in that Country.* By William Geddes, m.d., &c. &c., *late* Surgeon of the Madras European Regiment.—*London,* 1846. 8vo, pp. 492.

In the application of the numerical method to medical science, practical difficulties are encountered which, from their nature, must tend to throw doubt and uncertainty upon many of the results obtained. As one of the chief of these, we may notice the constant change of persons on whom the observations are made. In general practice an individual applies for advice, and after an attendance, perhaps of no great duration, recovers from his malady and is lost sight of; or, becoming discontented at what he considers the slow progress towards convalescence, politely bows out his medical adviser. Thus the subjects of observation are ever varying, and the effects of morbid action, or of therapeutic agents on each individual, are observed only for a short period. As a necessary consequence of this, the influence of age, temperaments, habits, and idiosyncrasies must constitute an important element in any calculation, and one of which it must be extremely difficult to form a just appreciation. Besides this, there is to be encountered the difficulty of ascertaining the previous medical history of the patient; who, sometimes from interested motives, and at others from ignorance, misleads the physician on the subject of former illnesses, which may, perhaps, bear an important relation to the origin or nature of the present attack. The uncertainty whether the prescribed medicines have been taken, the morbid love some patients have of "cheating the doctor," forgetful that they may at the same time be inflicting a serious injury on themselves, and the difficulty, frequently amounting to an impossibility, of enforcing dietetic regulations, and of restraining hurtful irregularities—all contribute to diminish the value of numerical observations when applied to medicine.

From most of these objections the public services are free. The military and naval surgeon has under his care, generally for a series of years, the same body of men, with the exception of such changes as arise from deaths, and the discharge of those who, old or disabled, are replaced by young men, selected as being apparently of sound constitution and in good health. These men are immediately brought before him when attacked by disease, which he has thus the opportunity of observing *ab initio;* he has also documents to which he can refer for the history of their previous illnesses from the period of their joining the service; he has the uncontrolled power of following out a particular plan of treatment, and the means of ascertaining that the medicine prescribed is administered, and that the regulations as to diet are rigidly enforced. He can also obtain pretty accurate information regarding their habits, and is sure that they are provided with those essentials to health—food, clothing, and lodgings. With all these advantages there are, even here, very peculiar circumstances to be taken into consideration, as exerting a powerful influence on the development and progress of disease; such as sudden change of climate, night duty, the aggregation of individuals in barracks and ships, diet, dress, drills, night watches, punishments, &c. &c. Nevertheless it is, we believe, the least objectionable field for observation, and one which we have long regretted to observe has been very inadequately cultivated.

The following observations, although now of very old date, bear so pertinently on this point in its relation to the naval branch of the public service, that we make no apology for extracting them. They form part of a paper "On the Practice of Medicine in the Royal Navy," written by the Editor of this Review, and published in the 'Edinburgh Medical and Surgical Journal,' for July 1810.

"It is a circumstance greatly conducive to his improvement that the young surgeon is appointed to small vessels, and that he is removed to a more extended practice as he has, or may be supposed to have, increased in knowledge. Among 70 or 120 sailors (the usual complement of such vessels) there will rarely occur so much sickness as might tend to perplex him; but he will have time thoroughly to study every case of disease that comes under his care. Unembarrassed by numbers and want of time, unseduced by any symptom, however prominent, and which might attract the whole attention of the cursory observer, he can leisurely and coolly review the whole phenomena of the disease, appreciate the value of the different symptoms, and thus be enabled to attain one of the most important of acquisitions to a medical man—a knowledge of nosology. Thus instructed, he will enter upon a larger field of practice with superior advantages; and, as he progressively arrives at greater heights, will be enabled to correct his speculations, and increase his knowledge by more extensive observation. The usual number of sick on board of our largest ships is sufficient to exercise the faculties of the experienced surgeon without fatiguing them.

"It cannot have escaped the observation of any one, who has witnessed the customs and habits existing in the navy, that the naval practitioner enjoys very superior opportunities for becoming acquainted with the laws which regulate health. Forming his observations on a limited body of men, leading the mechanical life of sailors, whose every action is public, the naval surgeon has it in his power to know the whole body of *juvantia et lædentia* that act upon their systems. He may not only be intimately acquainted with the *character* of every individual, but he may easily ascertain the quality, and even quantity, of every man's food and drink, the nature of his habits with regard to exercise, air, clothing, &c., from one year's end to another. The objects of his observations being placed, as it it were, on a stage, isolated in the middle of the ocean, and receiving, unadulterated, all the influences of the sky—it is in the power of the naval practitioner, I had almost said exclusively, to ascertain the real effects of the atmosphere on health. Living in comfortable apartments, with artificial means to counteract the season's temperature, and evade its violence, or placed in a thousand different situations, which modify or defeat its power, the inhabitants of the country or the city cannot be said to exhibit pure specimens of atmospheric influence. But the sailor, to whom no close apartments, or artificial heat, ever temper the season's rigour, or from whose head no roof or umbrella wards off the rain or sunbeam, must exhibit, in the conditions of his system, the real effects of the weather on health. And various, indeed, are the changes of the sky to which the British sailor is exposed.—

——— Quod mare Dauniæ

Non decoloravere cædes?

Quæ caret ora cruore nostro? Hor.

"In the actual treatment of disease also, we conceive the naval surgeon possesses some peculiar advantages; some relating to the patient's welfare, some to his own improvement. He sees the disease in its very commencement—a circumstance of great importance, both as tending to the discovery of the nature of the disease, as well as affording the best chance for curing it; for it is remarked that the progress of most diseases can only be stopped effectually in their earliest stages. In private practice the case is very different. During the cure the naval surgeon has the patient perfectly under his control. 'His proceedings being obstructed neither by the prejudices of ignorance, nor the weakness of affection,' he can employ and follow up the most vigorous and determined practice. And rarely, indeed, will he have to contend with the obstinacy of his patient, as frequently happens in private practice; for the simple and unpresuming seaman looks upon his medical attendant not merely as a surgeon, but, in implicitly following his directions, he not only thinks it reasonable to take the advice of the wiser man, but conceives it his duty to obey the commands of his officer. In domesticating with his patients, he can attend to every stage of the disease; is at hand on every emergency. Nor need he intrust the administration of medicines to an ignorant nurse; it is the duty of the assistant-surgeon to give them with his own hand. No food or drink can be given to the patient unknown to the surgeon. In short, as to what regards the treatment of the patient, the practice of the sick-bay is analogous to the practice of an hospital."

The Statistical Reports on the Health of the Army and Navy have put us in possession of an amount of valuable information on the influence of climate in producing disease and mortality among men in the prime of life, to which there is nothing similar in existence. But interesting and valuable as these documents are, they leave untouched the important subjects of symptomatology and therapeutics. The complex nature of the observations necessary for the elucidation of these two branches of science demands an amount of time and patient investigation which few medical men will be found able or willing to devote to it. We have great pleasure in directing the attention of our readers to the volume before us, as a proof that these interesting subjects have not been wholly neglected, and as a model for those who may be inclined to labour in this department. It is the production of an officer of the Honorable East India Company's service, and reflects the highest credit on his talents, industry, and judgment, while it also affords evidence of the fact that the splendid field for medical observation offered by India is being cultivated by labourers well qualified for the task.

In May, 1829, Dr. Geddes joined the Madras European Regiment, as surgeon, and remained in charge of it till the same date in 1833. This is an infantry corps, in the service of the East India Company, and is composed entirely of Europeans; the vacancies caused by death, or other casualties, being filled up by recruits from the United Kingdom. Immediately on his appointment he endeavoured to make himself acquainted, through the regimental records, with the history of each individual belonging to it, and afterwards kept careful notes of their diseases; which have furnished the materials for the volume before us. The object of the author has been to "afford a practical view of all the diseases with which a body of European soldiers has been affected within a certain period, and under usual circumstances, in the climate of the East Indies; the probable cause of their sickness; the treatment employed therein, and its results."

The regiment had been actively engaged in the Burmese war, from which it returned to the Madras Presidency in 1826. Many old soldiers were then invalided, and their places, as well as those of the men who died on service, were filled up by recruits from England; so that, in July 1828, "of 503 individuals present with the regiment, only 155 had been in a warm climate beyond three years." Although our author did not join the corps till May 1829, he has, by means of the official returns, extended his observations back to 1st July 1828, and included a period of five complete years, as far as relates to the prevalence of disease; but in his analysis of the cases he has confined himself to the period of four years, during which he was actually in charge.

Till the middle of December 1829, the regiment was quartered in the fort of Masulipatam, on the shores of the Bay of Bengal, situated about a mile and a quarter from the sea, on the northern bank of a salt-water creek. It is surrounded by a saline swamp, varying in breadth from 2000 to 3000 yards; beyond which an alluvial plain extends to a distance of about 40 miles. The regiment marched on the 15th of December to Kamptee, a distance of 520 miles, and arrived there on the 5th March 1830; having moved, on an average, about seven miles daily.

Kamptee is situated in lat. 21° 16′ N., long. 79° 46′ E., nine miles north of the city of Nagpoor, on the right bank of the river Kanan, at a height of 50 to 60 feet above its low water level. It is nearly equidistant from the eastern and western shores of the peninsula of India, and about 900 feet above the level of the sea. The soil on which the cantonment is built is of a clayey nature, but that of the surrounding country is chiefly a black alluvial deposit, termed cotton ground. The river runs over a sandy bed with considerable rapidity, is generally fordable, and its waters free from mud. The barracks were composed of several one-storied tiled buildings, raised a few feet from the ground, and sufficiently lofty and well ventilated.

The seasons here are divided into the hot, rainy, and cold: the first extending from the beginning of March to the beginning of June, the second from that period usually till October, and the cold season commencing in November, and terminating in the beginning of March. The period, however, of the commencement of each, and its duration, varies greatly in different years; the occurrence of the rains is the most important. "When they are heavy, or long continued, they have the effect of lowering the temperature at the time; of lengthening the duration of the succeeding cold season; and of diminishing the severity of the hot weather. They are no less influential in their effect upon the prevalence of disease. The epidemic constitution of the following year may be said, in usual circumstances, to have its origin in the seasonable occurrence, or otherwise, of the rains." In consequence of the influence of the rainy season, Dr. Geddes has dated the commencement of his annual returns from the 1st July in each year.

During the five years, from 1st July 1828 to 1st July 1833, the admissions into hospital averaged 2609 per 1000 of the strength annually, and the deaths 49·5, or, including those from accidents, violence, &c., and which did not come under medical treatment, 54 per 1000. As is generally the case in tropical climates, the admissions varied considerably in different years, the lowest ratio having been 2108 in 1829-30, and the highest, 3081, in 1831-2. In like manner, the deaths ranged between 27·6 and 66·6 per 1000.

If we compare the relative salubrity of the two stations, it appears that the admissions at Masulipatam, during 17 months, were in the annual ratio of 2499, and the deaths of 59·4, per 1000; while at Kamptee, in 40 months, they averaged 2755, and 44·2 respectively; but if we deduct from the latter the deaths by cholera, which did not prevail epidemically at Masulipatam during the period included in the 17 months, the ratio of mortality would be reduced to 34·8 per 1000. The regiment was very healthy during the three months occupied by the march, only 230 cases of disease having occurred, and 8 deaths, 7 of which were from cholera. Diseases of the bowels were considerably more prevalent and fatal at Masulipatam than at Kamptee; while, at the latter, the admissions from fever were much more numerous, but with a greatly lower ratio of mortality.

Dr. Geddes has given, in a tabular form, minute details of the composition of the regiment, in regard to the age of the men, their native country previous occupations, period of arrival in India, and length of service there; and has traced the medical history of each man during the five years included in his observations. He has also given monthly temperature tables for that period. Into these details we cannot enter at length; but must content ourselves with noticing some of the leading conclusions he has drawn, referring our readers to the work itself for the data on which they are founded.

Of the men composing the regiment on the 1st July 1828, or who joined it between that date and August 1829, 287 were English, 363 Irish, 42 Scotch, and 2 foreigners. During the five years under review, the mortality of the English and Irish was equal, being 25 per cent., while that of the Scotch amounted to 33 per cent.; but their numbers were too few to admit of any accurate deduction from this. Our author has classified the men according to their occupation previous to enlistment as follows: Labourers in the open air, 334; in house, 133; in constrained posture, 126; in heat, 42; simple confinement, 37; deleterious professions, 15; peculiar professions, 7. Among all these the mortality was nearly the same, from 20 to 24 per cent., except the labourers in a constrained position, or with exposure to heat, among whom it amounted to 32 per cent. in the five years.

From a careful consideration of the causes of admission into hospital of the young soldiers, Dr. Geddes comes to the conclusion that, in India, there is no acclimating fever.

"There is no disease of the nature of what has been named a seasoning one; which is usually understood to affect Europeans at some period shortly after their arrival in the warm climates of the West. It is true, indeed, that for the first few months of his residence in India, the young soldier is more frequently on the sick list than at an after period of his sojourn there. The diseases which are the occasion of this circumstance are, however, seldom of the nature of that which has received the name of a seasoning one; and may often be referred to causes connected with the drill necessary for the recruit, or with the change of habit and food consequent to his landing, after a long voyage, as a soldier, in a country so widely different from his own as that of India." (p. 78.)

After enumerating the diseases for which the men were first admitted into hospital after their arrival, he observes,

"Hence it appears that there is a greater proportion of local diseases, diarrhœa, and the class of peculiar complaints, among the earlier causes of admission to hospital than in future attacks of illness; that dysentery, indigestion, or syphilis are nearly equally numerous, either as first or after diseases; and that fever, hepatic disease, and rheumatism are more frequently met with after the patient has been once or oftener in hospital than among his earlier diseases." (p. 79.)

The annual ratio of admissions into hospital has been already stated to have been 2609 per 1000, which gives an amount of sickness a little above $2\frac{1}{2}$ admissions to each soldier annually; the average stay in hospital was $12\frac{1}{3}$ days, and the sick time, consequently, averaged 32 days to each man. The following table, which we have compiled from the work, shows how the admissions in each year were distributed, and has also an important bearing on the question of acclimatization, because it will be seen that, with the exception of 1832-3, which was remarkably healthy, the number of men who escaped disease entirely, diminished, while those who suffered under several attacks, increased in each succeeding year.

| Year. | Of the men composing the regiment there were in hospital during each year | | | | | |
|---|---|---|---|---|---|---|
| | Not at all. | Once. | Twice. | Thrice. | Four to seven times. | Eight times and upwards. |
| 1828-9 | 150 | 136 | 117 | 103 | 108 | 8 |
| 1829-30 | 145 | 130 | 121 | 94 | 109 | 2 |
| 1830-1 | 112 | 123 | 104 | 69 | 136 | 12 |
| 1831-2 | 83 | 92 | 95 | 88 | 148 | 20 |
| 1832-3 | 99 | 104 | 85 | 65 | 119 | 12 |

If, instead of each year separately, the whole period is taken, it appears that there were only four persons who had never been in hospital; 72

had been one to five times; 137 from six to ten; 107 from eleven to fifteen; 79 from sixteen to twenty; 31 from twenty-one to twenty-five; and 12 a greater number of times. "Of these, two were 44 and 45 times in hospital, and they (as well as in general the remainder of the 12) owed their frequent admissions upon the sick list to disorders the effect of their own imprudence, and chiefly of a trifling description." The average period between each admission of those who were oftener than once in hospital was 138 days, or about four months and a half.

A very important question, which has been frequently discussed in the army, is whether the age of recruits on their arrival in India exerts any influence on the mortality among them. The military authorities in this country being of opinion that very young soldiers were more amenable to fatal disease, ordered that no recruit for service in India should be enlisted under twenty years of age. Dr. Geddes gives the following as the result of his observations:

"In recruits, arriving before or at 20 years of age, the early deaths are comparatively few, apparently from the facility of change in the constitution at this period of life. The same is the case, although in a less degree, among those arriving in India about the ages of 24, 25, and 26 years; arising, it is believed, partly from the constitutions being somewhat more formed at this age than at 21, 22, or 23 years, while there is still some facility of change in it; and partly from there being, in general, greater steadiness of conduct in such individuals than among the younger recruits. A greater degree of mortality continued to show itself for a lengthened period among those coming out at the ages of 21, 22, and 23 years, than in the other two classes just mentioned; but after four years' residence in India, the proportion of deaths in those arriving at the ages from 24 to 26 inclusive, exceeded that of their earlier sojourn; while that of the youngest class became considerably less. The deaths among those men who arrived in India at a more advanced age than 26 are in greater proportion than among the younger soldiers. This is equally apparent at all periods of their residence in that country." (p. 83.)

We are by no means prepared to admit the perfect accuracy of this observation, because we believe the numbers to be too limited to counterbalance the influence of any accidental deranging circumstance; and we would require more extended evidence to convince us that the laws which regulate the increase of mortality with the advance of age in other countries are suspended in India. It, however, partially corroborates the observations which have been made of the comparative exemption from mortality of young soldiers, when not employed in the field, and militates against the recruiting regulation above noticed. The subject requires, and is deserving of further investigation.

Having thus briefly noticed some of the principal conclusions relative to the prevalence of disease and mortality generally, we shall now proceed to the consideration of our author's observations on particular classes of diseases. During the four years he was in charge of the regiment, 5517 admissions into hospital took place. The details of 4291 of these cases treated by himself were recorded by him at the bedsides of the patients, and have furnished the materials for the history of the different diseases.

I. Fevers.—1. *Intermittent and remittent.* From a careful study of this class of diseases in India, extending over a long series of years, during which he enjoyed peculiar opportunities of observing it, both among Europeans and natives, and in a great variety of circumstances, our author adopted the opinion of the identity of these two forms of fever; "apparently the produce of the same cause, showing similar symptoms, and removed by the same means, there seems no difference between these two forms of disease but that of severity."—He includes both, therefore, under the title of Paroxysmal Fever. In the four years from May 1829 to May 1833, during which he was in charge of the regiment, 1406 cases of paroxysmal fever were admitted into hospital, whereof 1210 were treated by himself, and on these, of which he kept careful notes, are founded his observations. From a table showing the monthly admissions of these cases, it appears that they were most numerous in August, September, October, and November, three fifths of the whole having occurred during these four months. Their numbers increase after the rains have set in, and are found to diminish again with the occurrence of the cold season, towards the end of which they reach the minimum.

Our author has gone minutely into the inquiry as to the proportion relapses bore to the original attacks, and the months in which they occurred. The 1210 admissions were confined to 405 individuals, and were thus distributed—119 had one attack in the four years; 111 had two; 59, three; 33, four; 25, five; 16, six; 17, seven; 14, eight; 6, nine; 2, ten; 2, eleven; and one soldier had 12 attacks. He has also stated in a tabular form the number of days between each attack. This depends a good deal upon the period of the year, the interval being shorter during the unhealthy than the healthy season. Thus, "of 495 cases of relapse which took place in the last five months of the year, 293 occurred within two months of the former attack; while of 310 relapses which were admitted into hospital during the remainder of the year, there were only 84 instances in which a longer period had not elapsed from the previous attack. Relapses, therefore, like the original attack are most liable to occur in the rainy season; and it appears that at this time the most usual period between each return of the disease is from 15 to 30 days; while a large portion of cases also take place either in a less interval, or after a longer period—on to two months." One half of all the relapses occurred within the last-named period.

From the tables illustrating the influence of age on the liability to paroxysmal fever, it appears that there is greater immunity above the age of 25, than among the younger soldiers. "Of 193 persons, who in 1830 were under the age of twenty-five, 100 were affected in the first year of their sojourn at Kamptee, and only 38 escaped altogether; while of 276 individuals at and beyond the age of twenty-five, 99 were affected in their first season at this station, and 81 had no fever previous to the period of the author leaving the regiment." This corresponds with the results of observations (which are, however, very limited) on the influence of age on fevers in temperate climates.*

The English suffered less than the Irish both in the number of attacks, and in the liability to relapse. The Scotch suffered less than either, but as already stated, their numbers were too limited to warrant positive deductions. With reference to previous occupation, those men who had been accustomed to simple confinement suffered least; next to these, were

* Thomson's Statistical Inquiry; Edinburgh Med. and Surg. Journal, No. 136; Fenger, on the Health of the Dockyard Workmen at Copenhagen; Annales d'Hygiène Publique, vol. xxiv.

labourers in the open air; while the class whose labour had been accompanied by exposure to heat furnished the greatest proportion.

Dr. Geddes makes a most important remark in regard to the health of the men who suffered from fever, which, however, we think requires confirmation from more extended observation.

"With the exception of the attacks of fever, the general state of health in those subject to this disease does not appear to differ very materially from that of the persons who had no fever. The average number of admissions within the five years, from July 1828 to July 1833, was, in the former, 12½, and, in the latter, 8½ times; the difference nearly corresponding with the average number of attacks of fever; while the average stay in hospital each time of being there, was, in those subject to fever, 11, and, in the others, 10 days. Of the more important diseases with which each class was affected besides fever, a slight predominance of hepatic disease and abdominal inflammation is found in the febrile subjects; while dysentery and rheumatism rather prevailed in the other." (p. 112.)

After a general description of the commencement and progress of paroxysmal fever, Dr. Geddes proceeds to show by numerical statements the relative frequency of the most common symptoms. He has given a table exhibiting the number of cases having headache, vomiting, or shivering, distinguishing first from subsequent attacks, and of which the general results may be thus stated. Shivering occurred alone in 94 cases, headache in 215, vomiting in 14; shivering and headache in 442, shivering and vomiting in 52, headache and vomiting in 87, and the whole three symptoms in 262; in 42 cases none of them were present, and 2 are stated as doubtful. Thus there was headache in 1006, shivering in 850, and vomiting in 415. Headache thus appears to be the most invariable of these symptoms.* It may occur at any stage of the disease; in a few instances where the febrile tendency had been checked by remedies, severe headache supervened during the accustomed period of fever, while the other indications of this state were either entirely wanting, or existed in a slight or partial manner. Vomiting, when present, usually occurs during the cold stage, and is generally accompanied by an irregular state of the bowels.

The state of the pulse being a point of much importance in fever, our author has shown in a tabular form, the variations during the first 48 hours after admission, obtained by recording the number of the pulse at two periods of the day. In 862 cases, the pulse fell to, or below 72; "in 237, it had been observed in that interval at from 90 to 100; in 265, at from 104 to 120; and in 43, at from 124 to 156, which is the highest number upon the record. Again in 317 of these cases the pulse was not observed at the usual visiting hours to be so high as 90, although at some stages of the disease it had, very probably, been beyond this number." "In 348 cases, the pulse ranged above 72 during the whole 48 hours immediately succeeding the patient's admission to hospital. In 126 of those, it had been observed at 76 within this period; but ranging in 56 of such cases to 100; in 58, from 104 to 120; and in the remainder above this number. In 179 cases, the lowest range of the pulse was 80, 84, or

* This is also corroborated by another table, showing the number of leeches employed in the fever, and the sites to which they were applied, from which it appears they were used in 849 cases, and in 763 of these were applied to the temples.

88; and of these, in 152, the highest range was 100 or beyond it. In 31 cases, the pulse ranged from 92 to 96; and in the remaining 12 from 100 upwards to 140. The lowest of these numbers are of course seen in the intermission or remission of the disease."—The pulse usually increases in frequency on the accession of the cold stage, reaches its maximum in the hot stage, and becomes softer, more full, and slower as the sweating stage advances.

With regard to the state of the bowels on admission to hospital, it appears that in 650 cases they were regular, in 344 they were more or less costive, and in the remaining 216 were in various states of looseness, from "open" to "dysenteric and vitiated." "Connected with the state of the bowels in fever, it is to be remarked that a call to stool is often an immediate precursor of the cold stage of the disease."

Our author considers at some length the various other symptoms, as pains in the limbs and back, in the region of the spleen and liver, at the scrobiculus cordis, &c; vertigo, tinnitus aurium, and deafness; the urinary and cuticular secretions, but into these details we cannot enter.

In a table, at page 136, are arranged the hours of attack in the cases of each type of paroxysmal fever, and distinguishing between first and subsequent attacks. When there was more than one attack, the relapses were generally of the same type, and occurring at the same period of the day as the first. The following table shows the hours of accession in each type. We have subjoined the results of some observations on the same point made at Blidah, in Algeria, in 1842 by M. Finot, Physician in Chief of the military hospital there.*

| | Quotidian | Tertian. | Quartan. | Double Tertian. | Total. | Military Hospital at Blidah. | |
|---|---|---|---|---|---|---|---|
| | | | | | | Quotidian | Tertian. |
| Before 7 A.M. | 14 | 70 | 0 | 46 | 130 | 260 | 173 |
| From 7 to 8 | 9 | 60 | 0 | 28 | 97 | 74 | 71 |
| 8 to 9 | 25 | 65 | 0 | 34 | 124 | 125 | 71 |
| 9 to 10 | 38 | 95 | 0 | 57 | 190 | 155 | 72 |
| 10 to 11 | 34 | 81 | 1 | 44 | 160 | 191 | 118 |
| 11 to noon | 41 | 52 | 1 | 27 | 121 | 120 | 63 |
| Noon to 1 P.M. | 52 | 25 | 1 | 5 | 83 | 209 | 75 |
| 1 to 2 | 39 | 21 | 1 | 3 | 64 | 64 | 32 |
| 2 to 3 | 34 | 12 | 5 | 4 | 55 | 112 | 47 |
| 3 to 4 | 33 | 8 | 0 | 2 | 43 | 73 | 33 |
| 4 to 5 | 22 | 8 | 1 | 0 | 31 | 65 | 23 |
| After 5 | 71 | 9 | 3 | 5 | 88 | 404 | 132 |
| Irregular or doubtful | 9 | 9 | 0 | 6 | 24 | 0 | 0 |
| | 421 | 515 | 13 | 261 | 1210 | 1852 | 910 |

From this table it appears that the accession of tertian fever generally took place at an earlier period of the day than the quotidian. Thus 161 attacks of the latter occurred before, and 260 after noon; while of single and double tertians 659 took place in the former, and only 117 in the latter period. M. Finot's observations furnish the same result, although the difference is not so strongly marked. Of quotidians, 925 attacks

* Mém. de Médecine Militaire, vol. lvi.

came on before mid-day, and 927 after that hour; while the paroxysms of tertian fevers were relatively 568 and 342.

In further illustration of the phenomena of paroxysmal fevers, Dr. Geddes gives an elaborate table showing—1st, the duration of the illness of the patient previous to admission into hospital; 2d, the number of paroxysms reported to have occurred during that period; 3d, the number observed while in hospital; and 4th, his stay there; distinguishing also the different types of fever, and the influence of the seasons upon these events. The first part of the table corroborates what we have already stated to be one of the advantages possessed by military surgeons, that of seeing disease in its earliest stages. Of 1197 cases (the total admissions exclusive of quartans), 225 came under treatment on the day of attack, 224 after one day's illness, 241 after two, 302 after three days, and the remainder after longer periods. This is still more marked if we consider the number of paroxysms before admission. In 8 there were none; in 436 there had been one; in 459 two; in 182 three; and in 112 only had more than three occurred. A greater proportion of quotidians and double tertians was admitted within the first 24 hours, than of the single tertians; and the men generally reported themselves sick earlier in cases of relapse, and at the season when fevers were most prevalent and severe, than in first attacks and during the healthy period of the year. The table also affords evidence of the benefit arising from this opportunity of early treatment, 676 cases having had no paroxysm after admission; 415 only one; 77 having had two; 17, three; and only 12 more than that number.

The stay in hospital depended in a great degree on the number of paroxysms after admission, the patient being generally detained three or four days after the last occurred. 883, or nearly three fourths of the whole, were discharged from the third to the sixth day; 226 were seven and eight days in hospital; and 88 for a longer period, chiefly from the disease being attended with severe complications, or inducing debility from its violence. The seasons did not appear to exercise much influence on the duration of the stay in hospital.

On the diagnosis of paroxysmal fever our author observes—

"It is not, in general, attended with much difficulty, when the symptoms are accurately recorded at two periods of the day, and due attention is paid to the nature and history of any attendant local affection. There are three classes of disease, however, for which it is liable to be mistaken: one, where a febrile state is the predominating complaint; another, where any of the common topical accompaniments of paroxysmal fever exist, and are attended by pyrexia; and the third, where a fever is present, putting on the paroxysmal form, but having its origin in some organic visceral disorder. In the first class are comprehended those ephemeral attacks arising from excitement by heat, drinking, and the like, or from disorders of the chylopoietic organs, or exposure to cold and moisture; in the second are comprised certain cases of cephalic or splenitic inflammation, or rheumatism, and cholera—all of which, when paroxysmal fevers are numerous, may put on so much the appearance of these fevers as to be confounded with them; and in the third are included cases of hectic fever, arising from hepatic abscess, phthisis, or other extensive organic lesions, producing constitutional irritation. In distinguishing such disorders from remittent or intermittent fevers, the medical attendant will be chiefly guarded by the prominence or presence of peculiar symptoms, or their more or less continued nature or duration; and it may be here remarked that nothing will be found to assist him more in arriving at a correct knowledge of the nature of the disease under treatment than accurate and minute records of the symptoms of the case, combined with the information which a series of such reports will afford respecting the previous history of the patient." (p. 149.)

Of the 1210 cases which have formed the subject of these observations, one only proved fatal. To illustrate, therefore, the manner in which paroxysmal fever terminates fatally, our author refers to the results of his experience on two different occasions when in charge of bodies of native troops. On the first of these, at Seringapatam, in 1823-4, of 1503 cases 23 died; and on the second, at Cuddapah, in 1826-7, of 955 cases 20 died. A tabular statement is given, showing the strength of the corps at Cuddapah, with details regarding the native countries of the men, the forms of fever, the average duration of the cases, the proportion of relapses, and the general plan of treatment. Into these particulars we shall not enter, but confine our remarks to the immediate cause of death in the fatal cases. In 14 out of the 20, repeated violent paroxysms of fever terminated in delirium or insensibility. In 8 this became superadded to the other symptoms in from two to five days after admission to hospital; in three, from six to nine days; and in three, after the ninth day from reporting themselves sick. "Among the first of these, death took place in three on the day following the occurrence of delirium or insensibility; in the same number the patient died on the second day afterwards; and in two, on the sixth and seventh days after. In the second class, the deaths were on the fifth and sixth days; and in the third, one took place respectively on the second, third, and fourth day after either of these symptoms had occurred. The cerebral affection was, for the most part, at first observed during an exacerbation of the disease." In three other cases death ensued from the violence of the febrile exacerbations, without the occurrence of stupor or delirium; in two, on the sixth day of the illness, and in the other, on the ninth. In another case, the patient, after two exacerbations, attended with slight delirium, and a tendency to vomiting and diarrhœa, died from "a sudden failure of the *vis vitæ* about the period of accession of the cold fit." In another case, dysentery supervened, and in the remaining one, an abscess formed in the site of an old injury of the chest, emptied itself through the bronchiæ, and the patient died of hectic fever. Of the 23 fatal cases at Seringapatam, 3 died in a state of delirium or stupor; 1 from a sloughing ulcer on the loins; 2 from the violence of the fever; 5 by the supervention of dysentery; 1 from beri-beri; and 10 from extreme debility, resulting from a chronic state of fever or of frequent relapses, with a tendency to œdema, or a loose state of the bowels. In one case the particulars are unknown.

We pass over our author's remarks on the causes of paroxysmal fever, and proceed to the consideration of the treatment. The indications which have directed the use of remedies in these cases were—"1st, to lessen the violence of the febrile paroxysm; 2d, to assist the tendency to an apyrexial state; 3d, to prevent the succeeding fit; and, 4th, to relieve local symptoms." In the cold stage increased covering, warm drinks, and a dose of opium, combined with calomel or antimonial powder, were employed; in the hot stage, bleeding, general or local according to symptoms, and antimonials, were the chief remedies; a dose of opium was also given with calomel, in cases where it had not been administered in the cold stage. When the fever began to decline, a purgative was administered if considered necessary. When the intermission took place, the medicine which was relied on to fulfil the third indication, that of preventing the succeeding fit, was the sulphate of quinine. This our author looks upon as a most invaluable remedy in such fevers, and, accordingly, gave it in all the cases except seven. He has entered into a very full detail of the amount given and the results obtained; and "the conviction produced on his mind by his whole experience is, that wherever a certain quantity of quinine can be exhibited in that period of a fever in paroxysms wherein the disease has declined from its acme, the tendency to a succeeding exacerbation will become checked, and all the other phenomena of the disease will of themselves, or by the use of minor remedies, disappear." In support of this opinion he adduces strong numerical evidence, by showing the number of paroxysms in each case, before and after admission. In one instance these amounted to 30 before admission, but in none did more than four occur after; in very few more than one; and in above half of the cases not even one occurred.

Our author has also carefully examined into "the nature and extent of those evil consequences which have been occasionally referred to the use" of this medicine. After stating the cases in which any of the unpleasant symptoms arising from it occurred, and tracing the subsequent history of the individual who laboured under them, he concludes, "it is not believed that any morbid effect likely to ensue from the use of this medicine, either immediately or after a lengthened period, need prevent its being given to the extent required for the removal of paroxysmal fever; and it is certain that any evil consequences which could possibly occur from its use are far counterbalanced by its immediate effects in checking the tendency to a recurrence of paroxysms; which effect cannot be secured so readily by any other method of treatment."

Holding this decided opinion in favour of quinine, our author preferred it, in every case, to giving mercury to the extent of affecting the mouth, which, in the earlier stages of his practice in India, was the treatment recommended in remittent fever, under the idea that when the system came under its influence, the febrile phenomena would cease to recur. Having tried this method in 87 of the cases at Cuddapah, already noticed, he has given a tabular statement of the results, and compared them with those obtained in his subsequent practice. From this table it appears

"That, commencing from the fifth or sixth day before the mercury took effect, the fever ceased to recur in an increasing number of cases each day, until that preceding the one in which the mouth became affected, when 19 patients got rid of their fever; that on the day in which the influence of mercury was observed the paroxysms of 9 ceased to recur; and that thence the numbers declined until the fifth and sixth days afterwards, at which time the cessation of the febrile paroxysms took place in three instances. The number of those altogether in whom the disease stopped before the affection of the mouth by mercury, amounted to 48, and of those in whom this circumstance took place after such an event, to 28. From these facts there is reason to doubt whether the mouth becoming affected is not rather a consequence of the cessation of the fever than the latter a result of the system having come under the influence of mercury; but in some chronic cases, where the contrary appeared to occur, an increase of frequency of the pulse, and of feverish irritation in the remissions, have been observed to take place in a gradual manner as the mercurial action showed itself, and this was considered to act by breaking in upon the habitual progress of the disease, which accordingly ceased to recur. In many instances, however, after a short interval of freedom from its attacks, these have returned before the affection of the mouth had entirely left the patient; and 37 of those who had been under the influence of mercury in the earlier months of the season had been seized with relapses before its expiration. From these circumstances, combined with a consideration of the occasional affection of the bowels, often amounting to a dysenteric state, produced by calomel, and of the lengthened sickness of the patient in consequence of his sore mouth, the reader will readily form an opinion of the relative value of mercury and quinine in putting a stop to that tendency to febrile exacerbations, which constitutes the main feature of the remittent and intermittent fevers of the East." (p. 189.)

The measures adopted to fulfil the fourth indication of treatment, that of relieving local symptoms, were, of course, various, depending upon the nature and severity of the complication. Great attention was paid to diet, both during the progress of the disease and in the stage of convalescence.

2. *Ephemeral and continued fevers.* These, in most instances, were obviously the result of the use, or rather the abuse, of intoxicating liquors. Occasionally they arose from exposure to the sun, or from errors in diet, and occurred chiefly among the young soldiers. The disease generally yielded to antiphlogistic treatment without much trouble.

Having thus placed before our readers some of the principal facts on the subject of paroxysmal fevers, we shall now proceed to examine briefly the observations on the other classes of diseases.

Diseases of the Head. In India these form a very important class of diseases, not as giving rise to a large amount of inefficiency from sickness, but on account of the very fatal character they assume, and their permanently disabling effects when death does not ensue. There is not much, however, in our author's observations demanding especial notice. The admissions from delirium tremens amounted to rather more than 7 per 1000 of the strength in the four years 1829 to 1833; which is a high proportion. This may, doubtless, be in some measure attributed to the pernicious system of the ration dram being still issued to the troops in India. A return of the quantity of arrack drawn on ration for the Madras European Regiment, from February 1830 to May 1833, inclusive, shows the quantity to have amounted to one twentieth of a gallon, or eight fluid ounces daily to each individual present with the regiment. The allowance of those in hospital, or of men disinclined to take it, was readily consumed by their comrades. In addition to this, there was a considerable quantity of spirits, arrack, wine, and beer sold in the canteen. "The quantity of intoxicating liquors thus used by the men would be considered great, if equally divided amongst them, and taken at divided intervals; but, in point of fact, while some soldiers observe great regularity in never exceeding their ration allowance, and a few restrict themselves to a less quantity, or even abstain from the use of arrack altogether for limited periods, generally under the sanctity of an oath, others, again, lose no opportunity of becoming intoxicated, and often retain themselves, more or less, in this condition for several days." With these facts before us, it is surprising that the disease did not prevail even to a greater extent; a result which may, perhaps, have been influenced by the large proportion of young men in the regiment; for it is well established that the effects of intemperance are developed in a rapidly increasing ratio with the advance of years.

The only points in connexion with this disease to which we shall direct attention, are the period that elapsed between the cessation of the drinking and the occurrence of the delirium, and the duration of the delirium in the cases which ultimately recovered. In 18 cases our author had an opportunity of noting accurately the first of these circumstances with the following results: In one instance the interval appears to have been 18 hours; in two, 24 hours; in two, 36 hours; in seven, 48 hours; in two, 60 hours; in two, 72 hours; and in two, 96 hours. "The author is strongly impressed with the idea that some period of a similar nature will be found to occur in all cases where the peculiar delirium of this disease manifests itself; or that where intoxicating liquors have been used on till its supervention, they have been taken in less quantity than at first, or if to an equal extent, their habitual use has rendered them less efficient in producing excitement of the sensorium." On the second point it appears that the length of time from the manifestation of the first symptoms of derangement until its finally leaving the patient was, in six instances, 24 hours; in seven, from one to two days; in three, from two to three days; in two, four days; in one, five days; in two, seven days; in one, eight days; in two, 11 days; and in one, 12 days.

The next group of diseases discussed consists of cases of

Thoracic Inflammation, comprehending, under this term, only pleuritis and pneumonia. Of these the details of but 42 cases are available for analysis, whereof three proved fatal; one having terminated in hydrothorax of the right side of the chest, and the other two in empyema of the left side. The number of cases is too small to admit of legitimate deductions from them. We, therefore, pass on to the next subject—the important class of hepatic diseases. Dr. Geddes has reserved the consideration of the cases of catarrh and phthisis for another occasion, but we may remark that of the latter, during the five years, July 1828-33, only two cases, one of which died, appear in the returns, out of an aggregate strength of 2687 men.

Hepatic Inflammation. During the five years reported upon by our author 280 cases of hepatic disease occurred, of which 21 proved fatal, being in the ratio of 104 admissions, and 7·8 deaths per 1000 of the strength annually.

After making some corrections, which are specified, the number of cases available for analysis amounted to 268, occurring in 141 individuals. These have been arranged under four heads: 1st, cases of abscess ending fatally; 2d, cases of probable abscess; 3d, acute attacks terminating in resolution; 4th, mild attacks with a similar termination.

1. *Cases of hepatic abscess terminating fatally*. During the four years, May 1829-33, 21 of the soldiers died from this disease, who furnished, with their previous admissions, 63 cases. To these our author has added in his analysis 10 who died previous to that date, but of whose cases accurate records had been kept. He has given very complete tabular statements of the leading circumstances of each case, and the principal appearances on dissection. The existence of hepatic abscess seems, in most instances, to have no connexion with the former illnesses of the patient. In 15 cases there had either been no admission previous to that indicating the formation of this disease, or their sickness had been very trifling, and quite distinct from it; in 9 the patients had been affected with dysentery; in 4, with rheumatism, 1 of whom had also splenitis; in 1, with jaundice; and 1 had, three years previously, been under treatment for catarrh.

"Upon the whole, with the exception of dysentery, and perhaps of rheumatism, it does not appear that abscess of the liver is often the consequence of any other disorder. Dysentery, too, is a very common symptom of hepatic abscess; and it is not improbable that in some of the cases registered thus, the hepatic affection may have already occurred, and proved in such cases a cause, and not a consequence, of the dysenteric disorder. The connexion, in short, between dysentery and hepatic abscess is such, that it is difficult in certain cases to say which is the original disease; and although due weight has been given to the attendant circumstances in forming a diagnosis, there has been a doubt—in arranging some of the dysenteric cases of patients where hepatic affection has afterwards become more decided—whether they should have been considered as idiopathic diseases or as symptomatic of the liver disorder." (p. 311.)

The nature and site of the abscesses in 29 of the fatal cases are thus briefly described:

"In 23 of the patients the disease consisted of one large abscess; in 3, of numerous small abscesses; in 2, of a combination of these, there being many small and one large abscess; and in 1 case there was one large abscess and another small one. The small abscesses have been found, generally, diffused through the whole substance of the liver; and when there has been a large abscess attending, this has been situated either in the middle or left lobe. In the case of two abscesses, the larger one was found in the right, and the smaller one in the left lobe. The greater proportion of the solitary abscesses was situated in the right lobe; there being twenty-one in this part of the liver, and only two in the left lobe. . . . . . Of those in the right lobe, 12 were seated in the upper part, near to the diaphragm, and in three of these the disease had communicated with the lungs, and been partly brought up by expectoration. In four the site of the abscess was more deep-seated, or in the posterior part of the liver. In one it was placed near its convex surface, and had been evacuated by an artificial opening in the right hypochondriac region; in another it was large and solitary, in the right lobe, but extending partly to the left; and in three the abscess was situated near the margin of the right lobe, which adhered in two of these cases to the colon. Of the solitary abscesses in the left lobe, one was situated near its concave surface, where it had burst into the cavity of the abdomen, and the other was placed in the upper part of the lobe." (p. 320.)

Our author divides the symptoms of abscesses of the liver into three classes: 1st, those more immediately resulting from the site of the disease, the chief of which is pain; 2d, those from sympathy with the affected organ, among which are dysentery, pain in the right shoulder, vomiting, and an altered or vitiated state of the bilious secretion; 3d, the effects of constitutional disturbance, generally in the form of hectic fever. From a table, showing the relative predominance of these at the earliest notice of the disease, it appears that pain was the most marked in 13 cases, dysentery in 10, and pyrexia in 7; but the degree in which these are experienced varies greatly in the course of the disease.

The great variety in nature and degree of the symptoms of hepatic abscess renders the diagnosis extremely difficult and uncertain. Of 31

fatal cases, only 19 had been entered in the returns as hepatic disease when they were admitted; and of the 42 admissions of the same individuals previous to the fatal attack, only 5 had been so entered.

"The remainder presented symptoms assimilating them, in twenty instances, to fever, either paroxysmal or continued; in seven to dysentery or diarrhœa; in one to cholera; in two to thoracic, and one to abdominal inflammation; in one to catarrh; in two to rheumatism; and in two to indigestion. . . . . . From these facts it will be understood that the symptoms arising from the site of the disease are not always found in a very marked degree. In some instances, indeed, they have been entirely wanting, and in others are found varying greatly in form and intensity, as well as in the period of the disease at which they make their appearance, and their continued nature when they do occur." (p. 322.)

Our author gives an abstract of the chief peculiarities observed in these cases with regard to pain, which presented great diversity in its seat, character, duration, and permanent nature. From this he draws the conclusion that, "although frequently indicative of the locality of the disease, it has, in some instances, been wanting altogether, or during certain periods of the complaint; in others it has been felt at a distance from the situation of the abscess; and it has occasionally shifted its chief site during the progress of the disorder. This symptom has also varied greatly in degree, as well as in its extent, and this independently of the size of the abscess."

The most conspicuous of the second class of symptoms, those, namely, from sympathy with the affected organ, was dysentery; which prevailed to a greater or less extent in almost all the cases at some period of their progress.

"These attacks, although occasionally distinguished by their tendency to continue upon the patient, were more remarkable—like some other symptoms in the progress of an abscess in the liver—by their disposition to relapse, the irregularities in their severity, and the slight and apparently disproportionate means by which they occasionally became ameliorated." (p. 351.)

Another symptom in the same class is pain in the right shoulder. With three exceptions, this was present at some period of the disease, when the abscess was seated in the upper part of the right lobe of the liver; but in none of those when it was at the lower margin, or in the left lobe, or where there were numerous abscesses. This fully corroborates the remark of Annesley, "that pain at the top of the right shoulder is, when present, certainly characteristic of the disease in the right lobe."

Vomiting, another symptom of this class, was seldom present when there was dysentery, with ulceration of the bowels. It was impossible to trace, in any marked degree, its presence or absence to the site of the abscess. "The size of this, in some measure, seems to operate in producing this effect, as the tendency to vomiting has in general become more urgent as the disease advanced; but its presence otherwise would appear mainly to depend upon the constitutional predisposition of the patient."

The last symptom of this class to which we shall advert is an increase, diminution, or vitiation of the bilious secretion. The last appears the most frequent consequence of hepatic abscess, the stools being altered in consistence, and almost always of an unhealthy colour, varying from a blackish or muddy hue to a bright yellow or dark green. Occasionally there has been a deficiency in the quantity of bile, while at other times it has been in excess. It does not appear that any of these peculiarities can be referred to the site of the abscess.

With regard to the constitutional symptoms, the fever, in most instances, assumes a paroxysmal character, although in a few it puts on more the appearance of inflammatory or continued fever. The pulse is generally recorded as thready or wiry, sometimes more or less sharp, irritable, or weak, and increasing in frequency as the case proceeds to a fatal termination. The tongue is generally furred; there is loss of appetite, with considerable thirst; and frequently want of sleep, or the sleep disturbed by unpleasant dreams. In one case only the abscess pointed externally, and was opened; in one, rupture took place internally, and the pus escaped into the cavity of the abdomen; and in three the abscess burst into the lungs.

*Of cases of probable abscess of the liver.* Under this head our author has included 34 individuals, who furnished 61 admissions from hepatic disease. From what has already been said of the difficulty of diagnosis, it is not surprising that we should entertain doubts whether abscesses really existed in some of these cases.

> "In the present section," says our author, "have been arranged those hepatic disorders which have exhibited similar symptoms [to those observed in the fatal cases comprised in the preceding section]; differing, however, from the former in the disease either becoming perfectly removed, or in its not proceeding to a fatal termination during the author's observation. Although varying in this respect, the similarity of symptoms, and in a few instances dissection, render it probable that they had owed their origin to the presence of the same cause; and that where the disease had disappeared entirely, this circumstance has been owing to the removal, in other words, to the absorption, or absence by some other means, of an abscess in the liver." (p. 365.)

Of the 34 persons furnishing the cases comprised under this head, seven died from other diseases, and the morbid appearances of the liver are stated in support of the accuracy of the classification; but we do not consider these to be satisfactory, at least in some of the cases. For instance, in one who died ten months subsequently to the hepatic attack, the appearances were, "in one spot, on the external surface of the right lobe, an indented mark, something like a scar, was observed, but not affecting the internal surface; the liver, generally, was somewhat smaller, paler, and softer than natural." In another, who died two years after being in hospital on this account, "the liver was irregularly indurated, of a pale colour; and on the outer surface of the right lobe there was a deep indentation, as of a scar, this penetrating the substance of the liver for some distance, in a tendinous structure." In another, "the liver was enlarged, and there was a spot on its convex surface resembling the cicatrix of an ulcer;" this man had survived the hepatic inflammation twelve months. In another, after nine months, "an adhesion was found of the liver to the ribs, with a white hardness on its surface, and the right lung had also formed adhesions to the pleura costalis." In a fifth, who died eighteen months afterwards of dysentery, "the state of disorganization was found to be such on dissection that it was difficult to say whether the adhesions of the colon to the concave surface of the liver, and otherwise, were owing to the recent disorder or to previous inflammation." In another case, sixteen months afterwards "the surface of the liver presented, in the upper part of the right lobe, a scar of an ulcer without any adhesions there, and the substance immediately below it was hard and white. The same appearance was presented on the edge of the right lobe, where there was a slight adhesion to the colon, and the whole of the liver was somewhat paler and softer than what is natural." In one case, where the patient died of dysentery four months after his last discharge for hepatic disease, two small abscesses were found. We do not consider these appearances to justify the inference that in all, or even most of the cases, there had been abscesses. That they were the result of inflammation we admit, but we are inclined to believe it was inflammation terminating in effusion of lymph, and not in suppuration.

The symptoms were of the same general character in these as in the previous class of cases, but milder in degree. The bowels were not so generally deranged, and when disordered usually presented the symptoms of diarrhœa rather than dysentery.

*Of acute hepatic inflammation ending in resolution.* Under this head are comprised 81 admissions into hospital, occurring in 41 individuals. These cases were of a more acute nature, and less complicated and obscure in their symptoms than those already considered. The chief characteristic symptom was pain in the side, varying according to the portion of the liver affected, and the extent of the inflammation. In six of these patients an opportunity occurred of examining the state of the liver at subsequent periods, and in all were found unequivocal traces of inflammatory action.

The symptoms in these cases resembled those in which abscesses were found. The bowels, however, were not often affected, and vomiting and derangement of the biliary secretion were rarely met with. The constitutional affection was usually of a severe description. The duration of the stay in hospital was in 19 cases less than ten days; in 24, ten to fifteen; in 14, fifteen to twenty; in 8, twenty to twenty-five; in 3, twenty-five to thirty; in 7, thirty to forty; and in 6 cases, upwards of 40 days.

*Mild cases of hepatic inflammation ending in resolution.* These, amounting to 63 admissions among 45 persons, were of the same nature as the preceding, but milder in their symptoms, and shorter in their duration. There is nothing in the details of them which demands our particular consideration.

In treating of the diagnosis of these affections, our author states the diseases which may be mistaken for the more obscure form of hepatitis to be "chiefly affections of the head of the colon, either from a morbid irritability or disease in that situation, or from a collection of flatus or fæces there, with or without disease of the colon. The symptoms in such cases have been observed to become very similar to those of hepatic inflammation; the pain occasionally extending to the right hypochondrie, and being attended with a greater or less degree of uneasy sensation in the right shoulder. Such cases seem chiefly to be distinguished by the original and principal place of pain being observed by the effect of pressure there, the degree of constitutional disorder, and the speedy benefit derived from purgatives or local applications. Some forms of dyspepsia may also be mistaken for inflammation of the liver. Pain of the contiguous parts produced by rheumatism, or by accident, or unusual exertion of the right arm, are likewise liable in India to be considered hepatic. In these and similar cases, or when the soldier feigns a pain in the region of the liver, the real nature of the complaint is most likely to be detected by a full consideration of the history and attendant symptoms, and by the effects of remedies."

In the treatment of hepatic inflammation the objects sought to be attained were—1st, to lessen the force of the circulation generally and locally; 2d, to excite another action more or less extensive; 3d, to remove peculiar symptoms; 4th, to restore the patient's strength after the disease had been subdued.

To fulfil the first indication venesection was employed, according to the urgency of the general disorder and the strength of the patient, followed by leeching, purgatives, and antimonial diaphoretics, and occasionally by poultices and fomentations to the part affected. For the second indication—the excitement of another action in the system generally—calomel, usually in combination with antimony or opium, was given as early as possible.

"It was believed, and this was confirmed by experience, that the specific action of mercury being excited in the constitution, the disease would give way to it, and the patient be left without any complaint but that produced by the effects of the medicine. In short, that simple inflammation and the action produced by mercury were incompatible in the same system; and hence that the sooner the latter took place, the more speedily the recovery of the patient would, in like manner, ensue." (p. 404.)

With the view of exciting a local action near the inflamed part, blisters were applied, and were found very useful in hastening the cure, and in saving depletion and consequent debility. The occasional symptoms demanding treatment were dysenteric or diarrhœal affection of the bowels, constipation, vomiting, and cough. With regard to these, the only peculiarity to be remarked was that the dysenteric affection occasionally produced by the use of calomel was almost invariably removed by a dose of castor oil, followed by an opiate. To accomplish the fourth object of treatment,—the restoration of the patient's strength,—nitrous acid, tonics, and, in protracted convalescence, wine, were given. When quinine was employed, its effects were closely watched, as it was supposed, where a tendency to inflammatory action remained, it might be renewed by a premature use of this remedy.

Such was the general line of treatment in the cases of inflammation, modified, of course, by the severity of the attack and the attendant complications, general bloodletting being less frequently necessary, and purgatives more trusted to, in proportion to the mildness of the disease. In cases of probable abscess, a local action was excited by means of blisters, which were kept open for some time, and counter-irritation by other means was also employed. Quinine was likewise given to check the disposition to fever, which, by producing debility, tended to diminish the powers of recovery. When suppuration was believed to have taken place, calomel was exhibited in smaller quantities than when the object was to remove inflammatory action. Our author believes the benefit in such cases to arise from the action of the mercury in exciting the absorbents to the removal of the purulent matter, and has found more benefit from it where only slight soreness of the mouth was produced than where this became severely affected.

Throughout the course of the disease, it is hardly necessary to observe, great attention must be paid to the diet of the patient. In the early stages this must be as low as possible, and afterwards the transition to his usual mode of living must be very gradual. Our author notices the advantage, in chronic cases, to be derived from a sea voyage, or even from change of residence.

"Many officers of the Indian army are annually sent on ship-board, suffering under symptoms of this disease; and of these a large proportion, by a voyage to Europe, or by proceeding to the Cape of Good Hope, New South Wales, or elsewhere, and a sojourn in those climates for certain periods, are eventually enabled to return to their duties free from complaint. A land journey, performed by regular marches, has occasionally been attended with the same effects." (p. 415.)

The next subject discussed includes a series of cases classed under the head of

Abdominal Inflammation, comprehending "all the instances of simple inflammation of any of the contents of the abdomen, with the exception of those of the liver," and the majority of which consisted of cases of splenitis. There does not appear to be anything connected with them which demands particular notice. Only one fatal case is included in this section, arising from peritonitis produced by rupture of the stomach, and escape of its contents into the abdominal cavity. The man, having been previously in good health, was suddenly seized with pain of abdomen, attended with vomiting, thirst, restlessness, and general soreness. The pulse became frequent, small, and contracted, the face collapsed, the cheeks sunk, cold sweats supervened, and he died in seventeen hours from the commencement of the attack.

"On dissection, a greenish, muddy, watery liquid was found in the cavity of the abdomen, with a few particles of some oil that had been given him three hours previously, floating in it. The whole of the intestines were glued to the omentum by coagulable lymph, and all these parts showed much vascularity. The external surface of the stomach also presented the same appearances; and it was found, on separating it from the body, that a small hole, round, and with even edges, penetrated its substance near the pylorus, and thence the liquid had escaped into the abdomen. This opening had scarcely an ulcerated appearance; and the inner surface of the stomach, as well as the intestines, was otherwise in a healthy state. The man had been nearly six years in India, had not been in hospital during the previous six months, and in none of his former illnesses had exhibited any symptoms which could be connected with the cause of his death." (p. 435.)

Rheumatism. During the five years 215 per 1000 of the strength were admitted annually into hospital affected with this disease. The seasons do not seem to exert any marked influence on it.

"Generally, it is to be remarked that neither the occurrence of heat, rain, or cold appears to have any reference to the prevalence of this disease. The following is the order in which each month ranges in respect to this circumstance: viz. July, December, April, October, May, June, August, September, February, March, November, and January; presenting in the first six months, or those in which it was most prevalent, the same number [of months] belonging to each season as in the healthy half year." (p. 443.)

During the period our author was in charge of the regiment 457 cases were admitted, the details of 312 of which, occurring in 160 individuals, were recorded by him, and form the subject of his remarks. Our space will not permit us to do more than advert to one or two of the more striking points. With regard to the seat of the disease—

"On a general review of the whole, 47 individuals are discovered to have been subject, either in their primary or relapsed attacks, to rheumatism both in the muscles and ligaments; 43 had those structures affected along with that of the periosteum; 28 and 23 had respectively disorders of the ligaments, or of the muscles only; in five individuals the disease was confined to the periosteum; in eight this structure was attacked with that of the joints, and in three with that of the muscles only. In the remaining three, the disease was chiefly of a neuralgic character, attended in two with rheumatism of the ligaments and muscles." (p. 444.)

The different parts of the body were affected in the following order of frequency: "the shoulders, particularly where both are seized, the knees in like manner, the loins and back, the thighs, one elbow, one shin, the hips, both or singly, the arms, ankles, back of the neck, and one heel, or both heels."

Dr. Geddes goes into minute details relative to the presence of fever in these cases, and its character, the number of relapses, the duration of the cases, and the period between each attack, with the apparent influence of native country and previous occupation on their prevalence. Into these particulars we do not intend to enter, but shall proceed to the consideration of the predisposing causes. In 52 individuals, or nearly one third of the whole, furnishing 117 cases, the disease was believed to have a syphilitic origin.

"In 13 of these persons the venereal affection had been confined to ulcers on the penis; in a like number there had been both ulcers and bubo, but the latter had not advanced to suppuration; in 7 the bubo had proceeded to this termination. In 19 cases the disease had been confined to one or more buboes, without any ulcer having been observed or recorded during the patient's stay in hospital; and in 10 of such cases the glandular affection had gone on to suppuration. In 19 of these 52 patients no mercury had been exhibited during the existence of the venereal disorder; in 4 this medicine had been given, but there was no affection of the mouth; in 9 it had produced a slight soreness of the gums; in 8, a greater degree of this; and in 12 cases the salivation was considerable. It is scarcely to be observed that the total absence of mercury, or its various degrees of exhibition or impression upon the system, had any effect in bringing on the rheumatic pains at an early period, nor does the nature of the venereal symptoms seem to exercise any influence in producing such result." (p. 450.)

Although in these cases syphilis appeared to be the predisposing cause, our author does not consider rheumatism to be a very general consequence of the disease.

"Of 180 individuals who came under treatment for venereal, 109 suffering under the different varieties of the complaint above mentioned, and apparently presenting no decided peculiarity, either in symptoms or treatment, different from those having rheumatism afterwards, were quite free from any attack of this disease.......... The general results would appear to be that, from constitutional peculiarities, the syphilitic virus gives a tendency to the occurrence of rheumatic inflammation; and it is probable that this disposition is increased by the exhibition of mercury, when the effects of this medicine are not so severe as to induce the patient to avoid exposure during the period of its being given to him.......

The only peculiarity in these cases was a greater tendency to affections of the small ligaments, as those of the hands and feet, or of the os sacrum; and to disorder of single joints, as of one elbow or one knee, than in other cases; while rheumatism of muscular parts, or of the same joints in both sides of the body at one time, are less frequent." (p. 451.)

The other classes into which our author divides the cases of rheumatism are, first, those in which the rheumatic diathesis appears to have become excited by a previous state of indisposition—as fever, dysentery, hepatitis, and, in a less degree, diarrhœa, cholera, and scrofulous tumours—aided by the exhibition of mercury. This comprises 31 cases, occurring among 18 persons. 2d. Those referrible to previous disease, like the preceding, but in which no mercury had been exhibited; and in this class are included 20 admissions among 11 persons. In the other cases of this disease no predisposing cause could be traced.

"In all classes the frequent recurrence of the disease, or its lengthened duration upon any one occasion, renders the patient pale, emaciated, and cripple; and other disorders, in some individuals, become generated, which eventually eclipse the original disease. The most common and formidable of these are ulcers on the skin, and certain symptoms referred to hepatic disease. By either, or as is usual, by both of these combined, or succeeding each other, rheumatism has occasionally proved fatal." (p. 472.)

Dr. Geddes gives an abstract of seven cases which terminated thus, and in six others it became necessary to discharge the men as unfit for service. In no class of diseases are the benefits of change of climate more strongly remarked than in this, the health being greatly improved, and often entirely restored, by a short residence in England.

We must now bring to a conclusion our account of the volume before us, which, as already observed, is most creditable to the author. We have endeavoured to lay before our readers a general idea of the contents of the work, but have been obliged to pass over many interesting points discussed in it, because, from the adoption of numerical statements, Dr. Geddes has given his observations in a form that scarcely admits of condensation, and which want of space prevents our giving at length. To the medical officers in India, and especially to those about to proceed thither, this will be found a valuable book of reference, and well merits to be included in the list of works with which officers are required to provide themselves on joining the service.

The author states in his preface that, should his health permit, and the present volume be favorably received, it is his wish and intention to extend his researches over the remaining diseases of which he has records in his possession, comprising, among other subjects, the very important class of diseases of the bowels. We sincerely trust he may be enabled to fulfil his intentions, and to complete a work which cannot fail to be productive of benefit to those in whose welfare, from his long association with them, he must feel a warm interest; and which will supply "to the experienced practitioner the means of knowing the success of certain methods of treating the diseases of India, and to the student of medicine a full and minute description of those diseases which he is most likely to meet with in the exercise of his professional duties in the East."

